# Germline Genetic Variants Disturbing the *Let-7*/LIN28 Double-Negative Feedback Loop Alter Breast Cancer Susceptibility

**DOI:** 10.1371/journal.pgen.1002259

**Published:** 2011-09-01

**Authors:** Ao-Xiang Chen, Ke-Da Yu, Lei Fan, Ji-Yu Li, Chen Yang, A-Ji Huang, Zhi-Ming Shao

**Affiliations:** Department of Breast Surgery, Cancer Center, and Cancer Institute, Department of Oncology, Shanghai Medical College, Fudan University, Shanghai, China; University of Washington, United States of America

## Abstract

Previous studies have shown that *let-7* can repress the post-transcriptional translation of LIN28, and LIN28 in turn could block the maturation of *let-7*, forming a double-negative feedback loop. In this study, we investigated the effect of germline genetic variants on regulation of the homeostasis of the *let-7*/LIN28 loop and breast cancer risk. We initially demonstrated that the T/C variants of rs3811463, a single nucleotide polymorphism (SNP) located near the *let-7* binding site in LIN28, could lead to differential regulation of LIN28 by *let-7*. Specifically, the C allele of rs3811463 weakened *let-7*–induced repression of LIN28 mRNA, resulting in increased production of LIN28 protein, which could in turn down-regulate the level of mature *let-7*. This effect was then validated at the tissue level in that the normal breast tissue of individuals with the rs3811463-TC genotype expressed significantly lower levels of *let-7* and higher levels of LIN28 protein than those individuals with the rs3811463-TT genotype. Because previous *in vitro* and *ex vivo* experiments have consistently suggested that LIN28 could promote cellular transformation, we then systematically evaluated the relationship between rs3811463 as well as other common LIN28 SNPs and the risk of breast cancer in a stepwise manner. The first hospital-based association study (n = 2,300) demonstrated that two SNPs were significantly associated with breast cancer risk, one of which was rs3811463, while the other was rs6697410. The C allele of the rs3811463 SNP corresponded to an increased risk of breast cancer with an odds ratio (OR) of 1.25 (*P* = 0.0091), which was successfully replicated in a second independent study (n = 1,156) with community-based controls. The combined P-value of the two studies was 8.0×10^−5^. Taken together, our study demonstrates that host genetic variants could disturb the regulation of the *let-7*/LIN28 double-negative feedback loop and alter breast cancer risk.

## Author Summary

Genetic variants are the genetic basis of inter-individual differences in susceptibility to common diseases like cancer. Single nucleotide polymorphisms (SNPs) are the major type of genetic variation in human beings, and previous studies have linked many SNPs with the risk of suffering human malignancies. In this study, we focused on microRNA (miRNA)–related SNPs and their relationship with the risk of breast cancer. MiRNAs are a kind of tiny endogenous RNAs that play important regulatory roles. *Let-7* is a miRNA that has been proven to be capable of inhibiting the tumor occurrence. LIN28, a gene that promotes cancer, is a known regulator of *let-7* and, interestingly, LIN28 itself is subject to repression by *let-7*. We have discovered that an SNP located near the target site of *let-7* in LIN28 disturbs *let-7*'s regulation of LIN28. This disturbance eventually leads to an increased level of LIN28 and a decreased level of *let-7*. We also, for the first time, confirmed that this SNP is associated with an increased risk of breast cancer in Han Chinese women. Our results suggest that genetic variants may potentially build a bridge from a physiological status to an aberrant, even pathological, status with the *let-7*/LIN28 loop in breast tissue, thus, facilitating carcinogenesis.

## Introduction

MicroRNAs (miRNAs) are an abundant class of ∼22 nucleotides (nt) long endogenous noncoding RNAs that can guide post-transcriptional gene silencing. They have important roles in multiple physiological processes, including developmental timing, cellular proliferation, differentiation, and apoptosis [1]. Deregulation of miRNAs has been linked with the occurrence, development, and prognosis of human cancers [2].

*Let-7* is a family of miRNAs that were initially identified to control heterochronic timing in the nematode *Caenorhabditis elegans* [3]. Defects in *let-7* in *C. elegans* result in unfavorable phenotypes, such as over-proliferation and lack of terminal differentiation, which resemble the characteristics of carcinogenesis in human beings. Subsequent studies revealed that *let-7* could regulate multiple oncogenes, such as RAS [4], MYC [4], [5], and HMGA2 [6]. A recent study has further linked abnormal *let-7* levels to the carcinogenesis of breast cancer and maintenance of malignant phenotypes [7]. Based on these findings, *let-7* is currently recognized as a type of tumor suppressor miRNA. LIN28, which was also initially discovered in *C. elegans* as a heterochronic gene [8], has been identified as a reprogramming factor that can induce pluripotent stem cells [9]. Overexpression of LIN28 promotes cellular transformation and is associated with advanced human cancers [10]. Taken together, these findings imply that LIN28 possesses oncogenic characteristics. Intriguingly, there is a double-negative feedback loop between LIN28 and *let-7*; *let-7* targets and represses the translation of LIN28 [11], [12], while LIN28 blocks the maturation of *let-7* miRNAs [13]–[16]. This cascade has recently been demonstrated to be involved in the oncogenesis of human cancers [17], [18]. It is therefore of critical importance that the *let-7*/LIN28 double-negative feedback loop is subjected to strict regulation to stay at an appropriate homeostatic status. Although detailed mechanisms of how this loop is regulated remain largely unknown, it can be deduced that slight changes in *let-7* or LIN28 levels can be amplified by the loop, resulting in more significant alterations. Theoretically, physiological variations in *let-7* and LIN28 levels are also subjected to the amplifying effect of the loop, leading to differential expression and/or function of these factors in different individuals.

One important source of inter-individual variation in phenotypes is genetic variants, the major component of which is the single nucleotide polymorphism (SNP). Many studies have shown that allelic variants of SNPs can influence the expression and/or function of their hosting genes. Several recent studies have further highlighted that the miRNA-related SNPs, especially those located within miRNA complementary sites, can remarkably alter the biogenesis and/or function of the corresponding miRNA [19]–[22].

Breast cancer is a common female malignancy, and the occurrence of breast cancer is associated with both genetic and environmental risk factors. Although previous studies have identified a group of high penetrance susceptibility genes and loci of breast cancer, such as BRCA1, BRCA2, and CHEK2 [23], these genes could only explain a small part of the genetic risk of breast cancer. In the past several years, SNPs have been widely employed as a type of genetic marker to identify susceptibility genes or loci of breast cancer. Using this strategy, many low penetrance susceptibility genes of breast cancer have been successfully identified.

These findings prompted us to investigate if there were any SNPs located with the *let-7* and LIN28 genes that could alter the biogenesis and/or function of these two factors and whether such genetic variants were associated with risk of developing breast cancer.

## Results

### Common genetic variants with potential functional effect in the *let-7* and LIN28 genes

We began by using *in silico* means to identify genetic variants located within the *let-7* and LIN28 genes that had potential functional effect. The 12 genes of *let-7* family with their 5′ and 3′ flanking sequences of 100 bp long as well as the coding sequence (CDS), promoter region, and 3′ untranslated region (3′ UTR) of LIN28 gene were searched in SNP databases as described in the Materials and Methods section. No common SNP that has minor allele frequency (MAF) higher than 5% in Han Chinese was found in the CDS of LIN28 and *let-7* genes along with their flanking sequences, indicating LIN28 and *let-7* are evolutionally conservative. We identified a SNP, rs3811464, in the promoter region of LIN28 that was 126 bp upstream of the transcriptional start site of LIN28. Although the initial prediction result showed that the minor allele of rs3811464 could lead to a loss of the binding site for one transcriptional factor named Zinc finger protein with Interaction Domain (ZID), a luciferase assay failed to confirm such an effect (data not shown). On the other hand, in the 3′ UTR of LIN28, we found three common SNPs (rs4659441, rs3811463, and rs6697410) among Han Chinese women. Because the 3′ UTRs are the primary binding and targeting site of miRNAs and recent studies have highlighted that allelic variants within the 3′ UTR could interfere with miRNA function [19]–[22], we then investigated whether these SNPs were located near the binding sites of miRNAs targeting LIN28. The predicted results showed that only rs3811463 was located near the target sites of several miRNAs (Figure S1A). Surprisingly and interestingly, the nearest miRNA target site to rs3811463 was that of *let-7* and, more importantly, this site was highly conserved across different prediction software packages. Therefore, we were very interested in whether allelic variants of rs3811463 could influence *let*-7-related regulation of LIN28 expression.

### *Let-7* differentially regulates LIN28 mRNA with allelic variants of rs3811463

**Figure 1:**
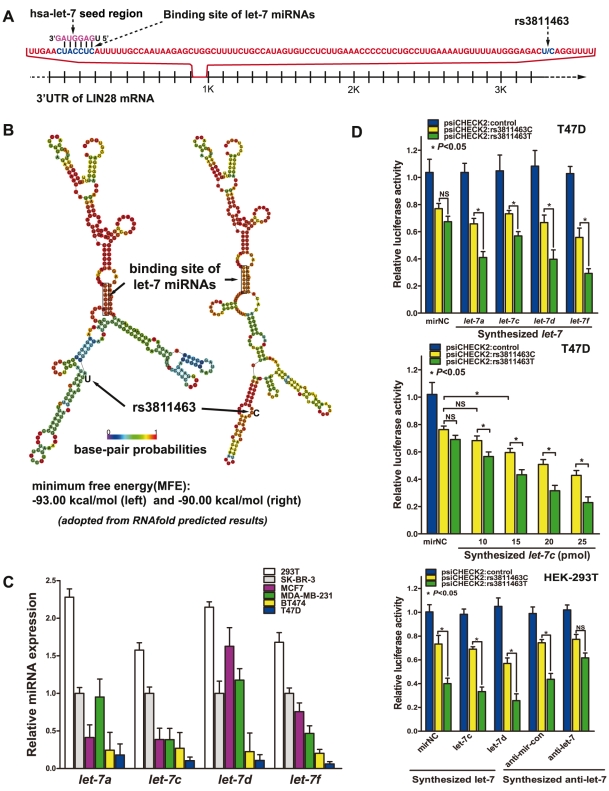
*Let-7* differentially regulates allelic variants of rs3811463. A, schematic representation of the *let-7* target site and the rs3811463 locus. A partial sequence of the 3′ UTR of LIN28 is shown. The rs3811463 SNP is located 81 nucleotides (nt) downstream of the “seed complementary sequence” of *let-7* in LIN28. B, the predicted secondary structure of the LIN28 mRNA. The secondary structures of the 3′ UTR of LIN28 were predicted by inputting two 301-nt long DNA sequences centering rs3811463 into RNAfold, with either the rs3811463-T (left) or rs3811463-C (right) allele. The figures and the values of minimum free energy (MFE) were generated by RNAfold (http://rna.tbi.univie.ac.at). C, levels of *let-7* (*let-7a*, *c*, *d*, and *f*) in different cell lines. The level of *let-7* was higher in HEK-293T cells than in several breast cancer cell lines, especially BT474 and T47D cells. D, luciferase reporter assays. The assays were performed in T47D and HEK-293T cells, which express low and high levels of *let-7*, respectively. Luciferase assays performed in T47D cells shows that overexpression of *let-7* can lead to differential suppression of the luciferase activity of psiCHECK2:rs3811463T and psiCHECK2:rs3811463C (top). By transfecting T47D cells with different amounts of *let-7c* mimics, we show that the differential suppression of psiCHECK2:rs3811463T and psiCHECK2:rs3811463C is in a *let-7* dose-dependent manner (middle). Compared with T47D cells, HEK-293T cells express relatively higher levels of *let-7*. This is why even when no *let-7* mimic is transfected into the cells (mirNC group), there are significant differences in the luciferase signal expressed by psiCHECK:rs3811463T and psiCHECK:rs3811463C (bottom, left groups). The *let-7* inhibition assay shows that inhibition of the endogenous *let-7* in HEK-293T cells is able to abolish the differences in the luciferase activity of psiCHECK2:rs3811463T and psiCHECK2:rs3811463C (bottom, right groups).

By comparing the genomic locations of rs3811463 and the binding site of *let-7*, we found that rs3811463 was located 81 bp downstream of the binding site of *let-7* in the 3′ UTR of the LIN28 mRNA (Figure 1A). Because the mRNA secondary structure is critical for mRNA-miRNA interactions [24], we first examined whether genetic variants of rs3811463 could alter the local second structure of the LIN28 mRNA based on the minimum free energy (MFE). RNAfold, an online RNA secondary structure prediction software, predicted that allelic variants of rs3811463 could alter the local mRNA secondary structure including that of the *let-7* binding site. The minimum free energy changed from −93.0 kcal/mmol to −90 kcal/mmol when the nucleotide at the rs3811463 locus changed from U to C (Figure 1B).

A luciferase assay was then performed to determine if *let-7* could differentially regulate LIN28 mRNAs with different alleles of rs3811463. To begin with, the baseline levels of the four representative *let-7* miRNAs (*let-7a*, *c*, *d*, and *f*) were evaluated in six common cell lines. The results revealed that two breast cancer cell lines, T47D and BT474, express relatively lower levels of *let-7* compared with other breast cancer cell lines (Figure 1C). Fragments of the 3′ UTR of the human LIN28 with either a T or C at the site of rs3811463 were then subcloned into the psiCHECK2 vector, generating two reporter vectors named psiCHECK2:rs3811463T and psiCHECK2:rs3811463C, respectively (Figure S1B). Different luciferase vectors (psiCHECK2, psiCHECK2:rs3811463T, or psiCHECK2:rs3811463C) were co-transfected with the synthesized human *let-7* mimics (*let-7a*, *c*, *d*, and *f*) or miRNA negative control (mirNC) into T47D cells. The dual luciferase assays demonstrated that artificial overexpression of *let-7* in T47D could lead to differential suppression of the luciferase activity of psiCHECK2:rs3811463T and psiCHECK2:rs3811463C; the psiCHECK2:rs3811463T showed significantly lower scores of luciferase activity when compared with psiCHECK2:rs3811463C. However, even though we observed significant differences between the luciferase signal expressed by psiCHECK2:rs3811463T and psiCHECK2:rs3811463C, we found that the luciferase activities of the psiCHECK2:rs3811463C vectors did not change much after transfection with synthesized *let-7* mimics, especially *let-7c* mimics. (Figure 1D, top). To determine whether *let-7* could regulate psiCHECK2:rs3811463C and the differential suppression of psiCHECK2:rs3811463T and psiCHECK2:rs3811463C was *let-7* dependent, we replicated the luciferase assay with increasing amounts of synthesized *let-7c*. The results showed that the luciferase signal of the control psiCHECK2 vector was comparable in each group regardless of the doses of transfected *let-7c* (data not shown). Both psiCHECK2:rs3811463T and psiCHECK2:rs3811463C vectors, on the other hand, were increasingly suppressed as the concentration of the *let-7c* mimics increased while psiCHECK2:rs3811463T was more significantly suppressed than psiCHECK2:rs3811463C (Figure 1D, middle). The same trend was observed when we replicated the assay with mounting amounts of synthesized *let-7f* (Figure S1C).

To further confirm that the differential suppression of psiCHECK2 luciferase vectors containing different 3′ UTRs of LIN28 was the specific effect of *let-7*, we performed a *let-7* inhibition assay. We first replicated the luciferase assay with different psiCHECK2 vectors and *let-7* mimics in HEK-293T cells. As indicated in Figure 1C, HEK-293T cell expresses a high level of endogenous *let-7*, which was reflected by the luciferase assay in that the luciferase activity of psiCHECK2:rs3811463T and psiCHECK2:rs3811463C showed a significant difference even when the cells were transfected with the negative control of miRNA mimic (mirNC) (Figure 1D, bottom). We then inhibited endogenous *let-7* (*let-7c* and *let-7d*) in HEK-293T cells using specific *let-7* inhibitors, the effects of which were confirmed by Taqman real-time PCR (Figure S1D). The luciferase assay showed that a reduction in the concentration of endogenous *let-7* in HEK-293T cells was able to abolish the differences in the luciferase activity of these two vectors compared with the baseline condition (Figure 1D, bottom), confirming that the differential regulation of psiCHECK2:rs3811463T and psiCHECK2:rs3811463C is the specific function of *let-7*.

These experiments showed that *let-7* could suppress expression of the luciferase gene by targeting the 3′ UTR of LIN28 and that the suppressing effect of *let-7* could be weakened when the nucleotide at the rs3811463 locus changed from the ancestral T to the variant C. This discovery indicated that T/C variants of rs3811463 could lead to differential regulation of the LIN28 mRNA by *let-7* miRNAs.

### LIN28 negatively regulates *let-7* in breast cancer cell lines

**Figure 2:**
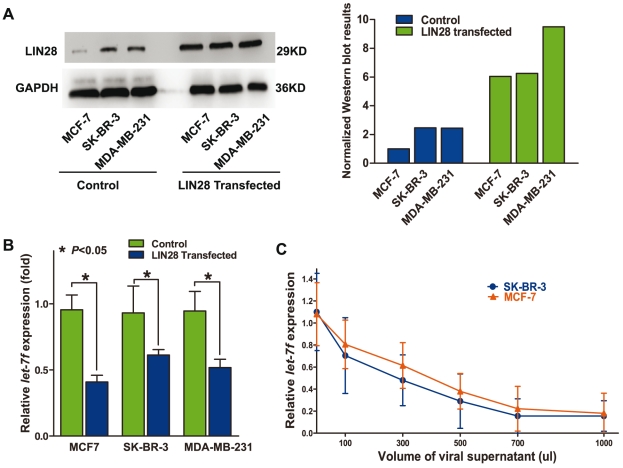
LIN28 represses *let-7* in the molecular context of breast cancer. A, overexpression of LIN28 protein in different breast cancer cell lines. The left panel shows a representative Western blot detection of LIN28 protein in MCF-7, SK-BR-3, and MDA-MB-231 transfected with control or LIN28 overexpression vectors, respectively. The right panel shows the quantified results of the Western blot assay. LIN28 is normalized to the loading control GAPDH. B, upregulation of LIN28 downregulates mature *let-7f* levels. The panel shows the levels of *let-7f* in MCF7, SK-BR-3, and MDA-MB-231 cells transfected with either control vector or LIN28-expressing vector. Significantly lower levels of mature *let-7f* were detected in cells overexpressing LIN28. C, the level of LIN28 is inversely correlated with *let-7f*. SK-BR-3 and MCF-7 cells were infected with increasing amounts of LIN28 vectors (determined by the volumes of the viral supernatant). The *let-7f* levels were evaluated 48 h after infection. As shown in the graph, there was an inverse dose-dependent relationship between LIN28 and *let-7f* levels.

Although previous studies have shown that LIN28 could negatively regulate *let-7*, it remains unknown whether this is the same case in the molecular context of breast cancer. To address this question, we arbitrarily overexpressed LIN28 in MCF7, SK-BR-3, and MDA-MB-231 cells, which express relatively higher levels of *let-7* (Figure 1C), and evaluated the change in *let-7* levels. Because the luciferase assay revealed that *let-7*f could more significantly repress LIN28 mRNA (Figure 1D, top), we took it as a representative of other members of the *let-7* family. Real-time PCR revealed that overexpression of LIN28 (Figure 2A) could lead to 30–60% lower levels of *let-7f* (P<0.05) (Figure 2B). To further investigate whether the reduction in the level of *let-7f* was LIN28-dependent, we infected SK-BR-3 and MCF7 cells with different volumes of unconcentrated viral supernatant containing LIN28 expression vectors. Real-time PCR results showed that there was a LIN28 dose-dependent reduction in the concentration of endogenous *let-7f* in SK-BR-3 and MCF7 cells (Figure 2C), proving that LIN28 could inhibit the maturation of *let-7* in the molecular context of breast cancer cells.

### Allelic variants of rs3811463 alter the levels of *let-7*, LIN28 mRNA, and LIN28 protein in normal breast tissue

Based on these findings, we proposed a biological model in which the variant rs3811463-C allele, compared with the ancestral rs3811463-T allele, can weaken the suppression of LIN28 mRNA by *let-7*, leading to overexpression of LIN28 protein, which in turn reduces the level of mature *let-7* and eventually results in altered status of the whole *let-7*/LIN28 loop.

To test this model, we randomly selected 300 patients who had donated both peripheral blood and normal breast tissue and determined their rs3811463 genotypes by RFLP-based assays. From these patients, we selected 30 patients with TT genotypes and 30 patients with TC genotypes. We did not include the rs3811463-CC genotype due to the rareness of this genotype. The included patients with the TT genotype were comparable to those with the TC genotype in regard to the distribution of breast cancer risk factors and the histological characteristics of their disease (Table S1).

**Figure 3:**
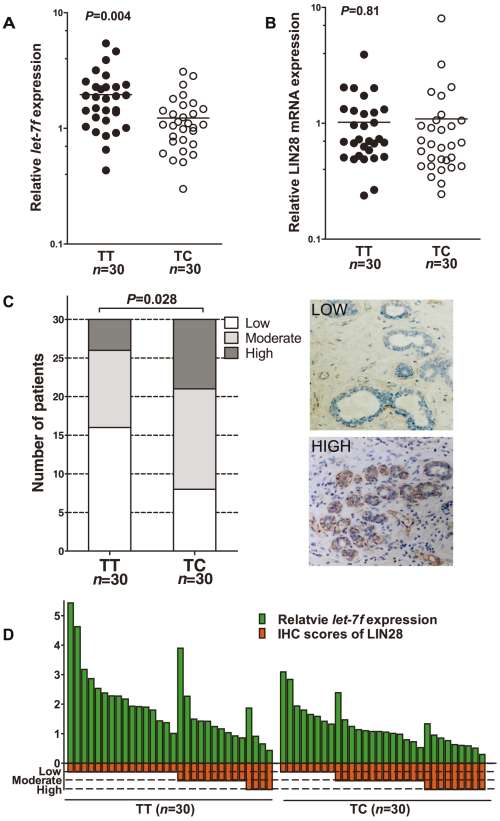
Differential expression of *let-7f*, LIN28 mRNA, and LIN28 protein in normal breast tissue of individuals with different rs3811463 genotypes. A, real-time PCR analysis of *let-7f* levels in RNA samples extracted from normal breast tissue donated by individuals with the rs3811463-TT genotype (n = 30) or rs3811463-TC genotype (n = 30). The horizontal lines represent the mean values. The mean *let-7f* levels in breast tissue of the rs3811463-TT genotype were approximately 1.6 times higher than in the tissue of the rs3811463-TC genotype (Mann-Whitney U-test, *P*-value = 0.004). B, real-time PCR analysis of LIN28 mRNA in the same batch of RNA samples. The horizontal lines represent the mean values. The mean LIN28 mRNA levels in the two groups were not significantly different (Mann-Whitney U-test, *P*-value = 0.81). C, LIN28 protein levels were investigated in the corresponding tissue with immunohistochemistry (IHC). The rs3811463-TC genotype was associated with increased levels of LIN28 protein as compared with the rs3811463-TT genotype (Cochran-Mantel-Haenszel χ^2^ test *P*-value = 0.028). The graphs on the right show representative IHC results. D, the relationship between *let-7f* levels and LIN28 protein expression levels in normal breast tissue. The graph depicts the levels of *let-7f* and LIN28 protein (IHC score) for each individual. Generally, lower levels of *let-7f* correspond with higher levels of LIN28 protein.

We then examined the levels of *let-7f*, LIN28 mRNA, and LIN28 protein in the epithelial cells of the acquired normal breast tissue. As shown in Figure 3A, a significantly lower level of *let-7f* was observed in the normal breast tissue of individuals with the TC genotype than in that of women with the TT genotype (*P* = 0.004). Although the LIN28 mRNA expression level was higher in the tissue with the TC genotype than in those with the TT genotype, the difference was not statistically significant (*P* = 0.81) (Figure 3B). However, the level of LIN28 protein was significantly higher in the breast tissue with the TC genotype than that with the TT genotype (*P* = 0.028) (Figure 3C). Furthermore, lower levels of *let-7f* generally corresponded to higher levels of LIN28 protein (Figure 3D), suggesting that *let-7* regulate LIN28 mainly through inhibiting the translation, rather than through inducing the degradation, of LIN28 mRNA.

Taking all these findings together, the rs3811463-C allele is correlated with a high level of LIN28 protein and a low level of *let-7* in a normal physiological context, which is consistent with our model.

### Two SNPs in LIN28, rs3811463 and rs6697410, are associated with breast cancer in the first case-control study

Our results thus far indicated that genetic variants in *let-7* and LIN28 genes, such as rs3811463, might potentially alter the levels of the *let-7*/LIN28 loop in individuals with different genetic backgrounds. Because the increased LIN28 levels and decreased *let-7* levels have been previously associated with carcinogenesis [7], [8], [10], we were interested in whether such genetic variants, especially rs3811463, were associated with the risk of breast cancer. Because an initial study failed to identify any common SNPs in *let-7* genes, we conducted association studies to investigate the relationship between common SNPs in the LIN28 gene (including rs3811463) and the risk of breast cancer at the molecular epidemiological level. The included SNPs were selected as described in the Materials and Methods section.

In the first hospital-based case-control study, we evaluated the frequency distribution of the selected SNPs in 1,004 cases and 1,296 controls. All genotyped SNPs had a minor allele frequency (MAF) greater than 9% (Table 1). Based on a threshold of 0.05, no significant deviation from Hardy-Weinberg Equilibrium (HWE) was found in the control group. As shown in Table 1, we found that two SNPs located within the 3′ UTR of LIN28 were significantly associated with breast cancer. Encouragingly, one of these two SNPs was rs3811463 and another one was rs6697410. The *P*-values continued to be significant after permutation tests or Bonferroni correction using a threshold of 0.05. When compared to the ancestral rs3811463-T allele, the rs3811463-C allele was associated with an increased risk of breast cancer (odds ratio (OR), 1.25; 95% confidence interval (CI) 1.06–1.47; *P* = 0.0091). In contrast, the rs6697410-G allele was associated with a reduced risk of breast cancer as compared to the ancestral rs6697410-T allele (OR, 0.76; 95% CI 0.63–0.93; *P* = 0.0068). The distribution of genotypes among cases and controls in the test set is shown in Table 2. Using the common homozygous genotypes as the references, we found that the rs3811463-TC genotype correlated with an increased risk of breast cancer while the rs6697410-TG genotype was associated with a decreased risk of breast cancer. However, significant associations between the homozygous genotypes of variant alleles (rs3811463-CC and rs6697410-GG) and the risk of breast cancer were not found, which might be due to the rareness of these two genotypes in the population.

**Table 1:** Frequency of LIN28 alleles in breast cancer patients and controls in the test set.

SNP	Position	Risk allele	Cases (n‡, %) Controls (n‡, %)		*P* §	*P* ∥
rs12122703(A>G)	c.−1645	A	1,711(85.9)	2,147(84.9)	N.S.	N.S.
rs3811464(G>A)	c.−240	G	1,702(85.2)	2,116(83.8)	N.S.	N.S.
rs11247955(G>A)	c.342+10135	G	1,783(90.2)	2,235(89.3)	N.S.	N.S.
rs3811463(T>C)	c.*993	C	319(16.2)	341(13.4)	**9.1×10^−3^**	**0.045**
rs6697410(T>G)	c.*3260	T	1,731(90.6)	2,239(88.0)	**6.8×10^−3^**	**0.037**

N.S., no significance. The significance of boldface is a *P*-value of <0.05.

**‡:** Number of allele.

**§:** Unadjusted *P*-value of two-sided χ^2^ test.

**∥:** *P* value after 1,000-permutation tests.

**Table 2:** Associations between LIN28 genotypes and breast cancer risk in the test set.

SNP	Genotype	Case (n)	Control (n)	OR (95% CI)*	OR (95% CI)†	Dominant model OR (95% CI)†, ‡	Recessive model OR (95% CI)†, ‡
rs12122703	AA	735	909	Reference	Reference	1.00 (0.81–1.23)	1.29 (0.69–2.43)
	AG	241	329	0.91 (0.75–1.10)	0.97 (0.78–1.21)		
	GG	20	26	0.95 (0.53–1.72)	1.28 (0.68–2.42)		
rs3811464	GG	725	882	Reference	Reference	0.83 (0.68–1.02)	0.86 (0.42–1.73)
	GA	252	352	0.87 (0.72–1.05)	0.83 (0.67–1.03)		
	AA	22	28	0.96 (0.54–1.69)	0.81 (0.40–1.65)		
rs11247955	GG	802	996	Reference	Reference	1.01 (0.80–1.28)	0.99 (0.36–2.74)
	GA	179	243	0.92 (0.74–1.13)	1.01 (0.80–1.29)		
	AA	7	13	0.67 (0.27–1.68)	1.00 (0.36–2.75)		
rs3811463	TT	693	951	Reference	Reference	**1.35 (1.10–1.67)**	1.20 (0.61–2.35)
	TC	265	297	**1.22 (1.00–1.47)**	**1.36 (1.09–1.69)**		
	CC	27	22	1.55(0.87–2.78)	1.29(0.66–2.54)		
rs6697410	TT	785	989	Reference	Reference	**0.75 (0.60–0.96)**	0.51 (0.21–1.26)
	TG	161	261	**0.78 (0.63–0.97)**	**0.78 (0.61–0.99)**		
	GG	9	21	0.54(0.25–1.19)	0.49(0.20–1.21)		

The significance of boldface is a *P*-value of <0.05.

*Unadjusted odds ratio (OR) and 95% confidential interval (CI) calculated by logistic regression.

**†:** OR and 95% CI calculated by logistic regression, adjusted for age, age at menarche, menopause status, number of birth, body mass index (BMI) and family history of breast cancer. Major genotype is indicated as reference.

**‡:** For the model, homozygotes for the major allele (1/1), heterozygotes (1/2), and homozygotes for the rare allele (2/2) are coded as a continuous numeric variable for genotype (i.e. 0, 1, and 2). The dominant model is defined as contrasting genotypic groups 1/1 versus (1/2+2/2), and the recessive model is defined as contrasting genotypic groups 2/2 versus (1/1+1/2).

### Validation of the test set results in a second case-control study

To validate our findings in the test set, we performed another independent case-control study involving 511 cases and 645 controls. Unlike the first study, all of the controls in the validation set were recruited from the participants of a community-based breast cancer screening program as previously described [25]. In this set, the results of the initial study were successfully replicated; rs3811463-C allele was associated with an increased risk of breast cancer, while rs6697410-G was associated with a reduced risk of breast cancer. The associations were more significant when the two studies were combined together; the *P* value for the rs3811463-C allele was 8.0×10^−5^, whereas it was 7.0×10^−4^ for the rs6697410-G allele. After adjusting for common breast cancer risks, these two allelic variants were still significantly associated with the risk of breast cancer (Table 3).

**Table 3:** Validation of the two susceptibility SNPs located in LIN28 in the validation and combined sets.

SNP	Allele or Genotype	Validation set		OR (95% CI)	*P*	Two sets combined		OR (95% CI)	*P*
		Cases (n, %)	Controls (n, %)			Cases (n, %)	Controls (n, %)		
rs3811463	T	812* (82.7)	1,105* (87.4)	Reference		2,463* (83.4)	3,304* (86.9)	Reference	
	C	170* (17.3)	159* (12.6)	1.46 (1.51–1.84)	**1.7×10^−3^** †	489* (16.6)	500* (13.1)	1.31 (1.15–1.50)	**8.0×10^−5^** †
	TT	342 (69.7)	487 (77.1)	Reference		1,035 (70.1)	1,438 (75.6)	Reference	
	TC+CC	149 (30.3)	145 (22.9)	1.46 (1.12–1.91)	**5.0×10^−3^** †	441 (29.9)	464 (24.4)	1.32 (1.13–1.54)	**6.6×10^−4^** †
				1.44 (1.08–1.91)	**0.01** ‡			1.36 (1.15–1.61)	**2.9×10^−4^** ‡
rs6697410	T	892* (90.3)	1,105* (87.6)	Reference		2,623* (90.5)	3,344* (87.9)	Reference	
	G	96* (9.7)	157* (12.4)	0.76 (0.58–0.99)	**0.04** †	275* (9.5)	460* (12.1)	0.76 (0.65–0.89)	**7.0×10^−4^** †
	TT	406 (82.2)	485 (76.9)	Reference		1,191 (82.2)	1,474 (77.5)	Reference	
	TG+GG	88 (17.8)	146 (23.1)	0.73 (0.54–1.00)	**0.05** †	258 (17.8)	428 (22.5)	0.75 (0.63–0.89)	**1.0×10^−3^** †
				0.69 (0.50–0.96)	**0.03** ‡			0.75 (0.62–0.90)	**2.0×10^−3^** ‡

The significance of boldface is a *P*<0.05.

*Number of allele.

**†:** Unadjusted *P* value of two-sided χ^2^ test.

**‡:** Adjusted for age and family history of breast cancer.

#### The distribution of rs3811463 genotypes among women with different molecular subtypes of breast cancer

Because a recent study revealed that *let-7* could regulate estrogen receptor (ER) alpha signaling [26], while our study showed that genetic variants of rs3811463 could alter the levels of *let-7*, we further evaluated the distribution of different rs3811463 genotypes among the different molecular subtypes of breast cancer. The breast cancer was divided into three subtypes according to the status of ER, PR, and HER2, as described in the Materials and Methods. There were 918 patients with IHC results for the three markers and rs3811463 genotyping information available. As shown in Table S2, the distribution of rs3811463 genotypes (TT *versus* TC+CC) in different breast cancer subtypes were not significantly different (*P* = 0.26).

#### LIN28 promotes transformation of a normal breast epithelial cell line

**Figure 4:**
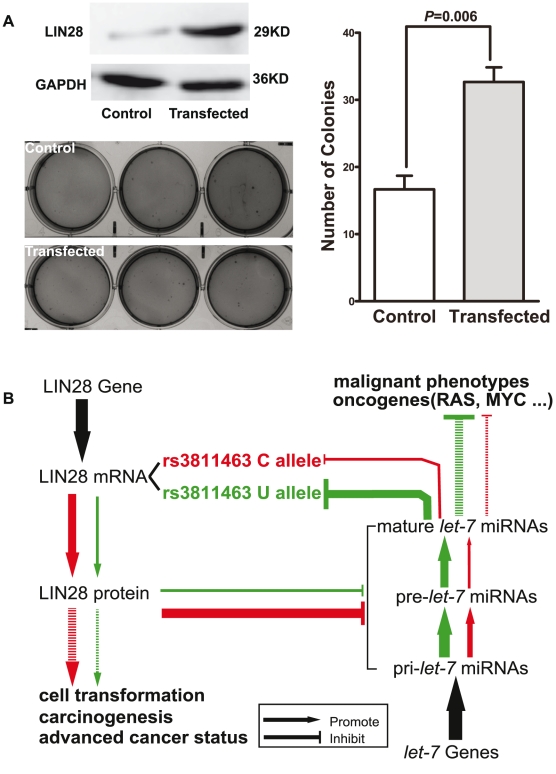
LIN28 transforms MCF 10A cells. A, LIN28 transforms MCF 10A cells. The upper left panel shows the Western blot analysis of protein extracted from the control MCF 10A or the LIN28-overexpressing MCF 10A cells. The bottom left and right panels show the number of colonies formed in the soft agar assay. Data shown represent mean colony numbers with error bars showing standard errors. MCF 10A cells stably overexpressing LIN28 formed significantly more colonies than the MCF 10A cells infected with control viral supernatant (*P* = 0.006). B, schematic representation of the biological model. In this model, LIN28 harboring the rs3811463-T allele was set as the baseline condition (green loop). Replacement of the T allele by the C allele at rs3811463 weaken the suppression of the LIN28 mRNA by *let-7*, leading to overexpression of LIN28 protein and, in turn, lower levels of *let-7*. Both high levels of LIN28 and low levels of *let-7* are associated with carcinogenesis, as indicated in the Introduction section.

Finally, we evaluated the ability of LIN28 to transform normal breast epithelial cells using the soft agar assay. MCF 10A cells infected with control viral vector and MCF 10A cells stably overexpressing LIN28 were cultured in soft agar medium under the same conditions for 3 weeks. As shown in Figure 4A, MCF 10A cells overexpressing LIN28 formed significantly more colonies than the MCF 10A infected with control vector (*P* = 0.006). These results suggest that LIN28 could promote the transformation of normal breast epithelial cells, which is consistent with previous studies [10]. This result, combined with the above-mentioned discovery that the rs3811463-C allele could weaken *let-7*-related suppression of LIN28 protein expression and lead to overexpression of LIN28, could in part explain the relationship between the rs3811463-C allele and the risk of developing breast cancer.

## Discussion

In the current study, we for the first time confirmed that the C allele of rs3811463, a SNP located near the binding site of *let-7* in LIN28, can weaken the suppression of LIN28 by *let-7*, disturb the *let-7*/LIN28 double-negative feedback loop, and ultimately result in an increased level of LIN28 protein and decreased level of *let-7*. Population-based association studies further related the rs3811463-C allele with an elevated risk of breast cancer.

*Let-7* is comprised of a family of miRNAs that can target and repress the translation of LIN28 mRNA into LIN28 protein [11], [12]. LIN28 protein, on the other hand, can block the maturation of *let-7* [11], [13], [15], [16]. Thus, these two factors form a unique double-negative feedback loop. *Let-7* is a recognized tumor suppressor [3], while LIN28 acts like an oncogene that can promote cellular transformation [8], [10]. Deregulation of both *let-7* and LIN28 has been associated with tumorigenesis [7], [10]. Accumulating evidence has also demonstrated that the *let-7*/LIN28 loop can interact with other factors, such as RAS, MYC, and NF-kB [17], [18], to form a complex network associated with carcinogenesis.

However, the mechanisms by which *let-7*/LIN28 loop homeostasis is maintained and whether inter-individual differences in the levels of this loop are associated with the risk of developing cancer, such as breast cancer, remain largely unknown. The results of this study suggest that germline genetic variants of LIN28, such as with rs3811463, may lead to altered status of the *let-7*/LIN28 loop and are associated with breast cancer susceptibility. As illustrated in Figure 4B, we define the *green region* as the normal condition, in which the nucleotide at the locus of rs3811463 is the ancestral T allele. Under this condition, *let-7* can sufficiently regulate LIN28, maintaining LIN28 protein at a relatively low level. The replacement of the T allele by the variant C allele, however, weakens the suppression of LIN28 mRNA by *let-7*, resulting in overexpression of LIN28 protein, which can in turn reduce the level of *let-7*. With the amplifying effect of the *let-7*/LIN28 double-negative feedback loop, the concentration of LIN28 protein will increase, while the level of *let-7* decreases until a new balance is achieved by mechanisms that remain unknown (Figure 4B; *red circle*). Both the increased level of LIN28 protein and reduced level of *let-7* are associated with carcinogenesis, which explains the relationship between the rs3811463-C allele and an increased risk of breast cancer, as shown by the case-control studies.

It should be noted that even though in this study we have proven that genetic variants of rs3811463 could influence the regulation of LIN28 by *let-7*, we cannot exclude the possibility that they can also interfere with other regulatory mechanisms. The first possibility is that genetic variants of rs3811463 can interfere the functions of other miRNAs besides *let-7*, especially considering that there are target sites for several other miRNAs near the binding site of *let-7* according to the target prediction results. This possibility is also demonstrated in the luciferase assays, in which the luciferase activity of both psiCHECK2:rs3811463T and psiCHECK2:rs3811463C were significantly lower than those of the control psiCHECK2 vector even when no synthesized let-7 was transfected, and the concentration of endogenous *let-7* is relatively low in T47D cells. A second possible mechanism is that the T/C variants of rs3811463 might alter the functional motif of other regulatory factors apart from miRNAs, even though there is currently insufficient evidence for this possibility. In addition, even though we have demonstrated that T/C variants of rs3811463 can lead to differential suppression of LIN28 mRNA by *let-7*, we are still unclear about the precise mechanism of how these genetic variants influence the function of *let-7*. Thus, more studies are warranted to elucidate these questions.

The occurrence of human breast cancer is generally attributed to both the genetic and environment risk factors. Much effort has gone into identifying the genetic risk factors of breast cancer and previous studies have found a group of high-penetrance susceptibility genes for breast cancer, which are generally involved in DNA repair pathways. Mutations in these genes are rare but have significant effects [23]. However, these genes only account for a small proportion of the genetic risk of breast cancer. It has become increasingly evident that common low-penetrance genetic variants may have a more prominent role [27]. SNPs are the most common type of genetic variants in human genome [28]. They are the genetic basis of the inter-individual variations in heritable phenotypes, such as susceptibility to cancers. A number of SNPs have been identified by previous studies to be associated with breast cancers, many of which are located within the promoter or CDS regions of genes [25], [29]. Our study, on the other hand, focused on miRNA-related SNPs. This study was part of a research program that aimed to identify: 1) common SNPs located within miRNA genes, miRNA target sites, and miRNA regulatory genes; and 2) the relationships between these SNPs and the risk of breast cancer. It was previously hypothesized that miRNA-related genetic variants might alter the biogenesis and/or function of their corresponding miRNAs [30], [31]. This hypothesis has been validated by multiple studies that found that SNPs located within or near the target sites of miRNAs could influence the functions of miRNAs and were associated with human cancers [19]–[21]. It seems that this is a common mechanism [22]. In this study, we demonstrate that the genetic variants of rs3811463, a SNP located within the 3′ UTR of LIN28, can weaken the suppression of LIN28 by *let-7* and is associated with the risk of breast cancer. Our study and previous studies all suggest that miRNA-related SNPs, especially those located near the complementary sites of miRNAs, are a new source of human cancer susceptibility loci.

In the case-control studies, we also found that another SNP, rs6697410, was associated with the risk of breast cancer. The underlying mechanism is still to be elucidated. *In silico* analysis showed that rs3811463 was located at the end of the 3′ UTR of LIN28, and there was no miRNA target site nearby. We propose several possibilities. First, it is possible that other polymorphisms linked with rs6697410 are responsible for this relationship, which means that rs6697410 is a marker for another functional SNP rather than a functional SNP by itself. Second, the nearby sequence may contain target sites for certain miRNAs, but the current *in silico* analysis tools failed to identify them. Third, genetic variants of rs6697410 may exert their function by influencing other function motifs involved in the regulation of LIN28 [32]. Further studies are needed to address this problem.

Finally, the focus of the current study is on the influence of genetic variants on the phenotypic variations of the *let-7*/LIN28 loop and their impact on the risk of developing breast cancer among women with different genetic backgrounds. We found that the existence of the *let-7*/LIN28 double-negative feedback loop might amplify even slight variations in the levels of *let-7* and/or LIN28, which could result in significant biological or even pathological effects. Based on such a finding, we hypothesize that other mechanisms that also change the levels of *let-7* and/or LIN28 are equally possible to induce the same effects. These mechanisms may include an epigenetic switch, transcriptional regulation, and so on. In addition, we are also aware of the deficiencies inherent in the design of our study: the possibility that we may miss genetic variants with low allele frequencies but prominent function. Even though we have 95% power to identify SNPs like rs3811463 (MAF = 14.6% and OR = 1.31), when we combine the two studies together, the power to detect those with MAF less than 10% drops dramatically to approximately 75%. Thus, further investigations are warranted to fully evaluate the effects of other possible mechanisms as well as the effects of the less common SNPs in the LIN28 and *let-7* genes.

## Materials and Methods

### Identification and *in silico* analysis of genetic variants involving the *let-7*/LIN28 loop

The miRBase, the Gene and dbSNP databases of National Center for Biotechnology Information (NCBI), and the International HapMap Project database were first employed to identify SNPs located within the LIN28 and human *let-7* genes that had potential functional effect. The coding sequence (CDS), promoter region (defined as the 2 Kb sequence upstream of the transcriptional start site of LIN28), and 3′ untranslated region (3′ UTR) of LIN28 were located using Gene database of NCBI. The genes of human *let-7* (*hsa-let-7*) were determined through data curated by miRBase and confirmed through Gene database of NCBI. Because the *let-7* genes acquired from miRBase and NCBI actually are those encode the precursors of *let-7*, we further identified the 5′ and 3′ flanking sequences of 100 bp long of these genes. SNPs located within the above-identified sequences were searched in the dbSNP database of NCBI and International HapMap Project database. The criteria for SNP selection was based on: 1) minor allele frequency (MAF) higher than 5% in Han Chinese in Beijing (CHB) according to HapMap database in order to be appropriate for including in the following case-control studies and 2) have potential functional effect based on the *in silico* analyzing methods as listed below. TESS (http://www.cbil.upenn.edu/cgi-bin/tess/tess) and TFSEARCH (http://www.cbrc.jp/research/db/TFSEARCH.html) were used to analyze the binding sites of the transcription factors. TargetScanHuman 5.1 (http://www.targetscan.org/), PicTar (http://pictar.mdc-berlin.de/), and MicroCosm Targets Version 5 (http://www.ebi.ac.uk/enright-srv/microcosm/htdocs/targets/v5/) were employed to predict the target sites of miRNAs. RNAfold software (http://rna.tbi.univie.ac.at/) was used to calculate the secondary structure of RNA based on minimum free energy (MFE).

### Luciferase reporter assay

An 885-bp fragment of the 3′ UTR of human LIN28 gene centering rs3811463 and the predicted complementary site of *let-7* was subcloned into the psiCHECK2 vector (Promega, Madison, WI, USA) using the Xhol and NotI restriction sites located 3′ to the *Renilla* luciferase translational stop codon. A mutant plasmid with C allele at the site of rs3811463 was constructed using a site-directed mutagenesis kit (Stratagene, La Jolla, CA, USA). All vectors were verified by direct sequencing. The generated reporter vectors were named psiCHECK2:rs3811463T and psiCHECK2:rs3811463C, respectively.

To conduct the luciferase reporter assay, T47D and HEK-293T cells were cultured in 96-well plates at 4,000 cells/well. After an overnight incubation, each well was treated with a transfection mixture consisting of 150 µl of Opti-MEM (Invitrogen, Carlsbad, CA, USA), 0.75 µl of Lipofectamine2000 (Invitrogen, Carlsbad, CA, USA), 10 pmol of synthesized miRNA [negative control (mirNC) or let-7 mimics (*let-7a*, *c*, *d*, or *f*) (GenePharma, Shanghai, China)] and 0.1 µg of psiCHECK2 vector (empty, psiCHECK2:rs3811463T, or psiCHECK2:rs3811463C). After a five-hour incubation, medium in each well was replaced by 150 µl of serum-containing medium. After 48 h, the *Renilla* and firefly luciferase activities were measured by the Dual-Luciferase Reporter Assay (Promega, Madison, WI, USA) on a Veritas Microplate Luminometer (Turner BioSystems, Sunnyvale, CA, USA) according to the manufacturers' protocol.

To determine whether the differential suppression of the luciferase activities of psiCHECK2:rs3811463T and psiCHECK2:rs3811463C was *let-7* dependent, similar luciferase reporter assay was performed in T47D cells with mirNC and different doses of synthesized *let-7c* (5 pmol–25 pmol) or *let-7f* (10 pmol–20 pmol). The *let-7* inhibiting assay was performed by transfecting HEK-293T cells with a transfection mixture consisting of 150 µl of Opti-MEM (Invitrogen, Carlsbad, CA, USA), 0.75 µl of Lipofectamine2000 (Invitrogen, Carlsbad, CA, USA), 20 pmol of *let-7* inhibitors [negative control (anti-mir-con) or anti-*let-7c* plus anti-*let-7d* (10 pmol, respectively) (GenePharma, Shanghai, China)] and 0.1 µg of different psiCHECK2 vector (empty, psiCHECK2:rs3811463T, or psiCHECK2:rs3811463C). The remaining procedures were identical to those of the above-mentioned luciferase assays.

Each experiment was performed at least three times in triplicate. The luciferase score was calculated by normalizing the luciferase signal of *Renilla* to that of firefly. Fold change (relative luciferase activity) was reported by setting the scores of the control groups as one and normalizing the scores in other groups.

### Acquisition of normal breast tissue, real-time PCR, and Western blot analyses

Normal breast tissue was donated by patients who had undergone a mastectomy in the Department of Breast Surgery after completing a written informed consent document. The fresh breast tissue was obtained immediately after surgery, snap-frozen in liquid nitrogen, and stored at −80°C. Careful dissection and collection of normal epithelial cells from the fresh breast tissue was achieved by employing laser-capture microdissection (Veritas Automated Laser Microdissection System) as previously described [33].

Total RNA was extracted from cultured cells or derivatives of fresh breast tissue using TRIzol (Invitrogen, Carlsbad, CA, USA) and cDNA was synthesized with the RevertAid First Strand cDNA Synthesis Kit (Fermentas, Vilnius, Lithuania) according to the manufacturers' protocols. Real-time PCR evaluation of LIN28 mRNA was performed using the SYBR Green fluorescent-based assay (Takara Bio, Shiga, Japan) in triplicate as previously described [25]. GAPDH mRNA was used for normalization. The small RNA was isolated and enriched using the mirVana miRNA isolation kit (Ambion, Austin, TX). RNA (10 ng) was used for synthesizing cDNA of *let-7* and U6 control using the reverse transcription (RT) primers (Applied Biosystems, Foster City, CA, USA). The RT was performed with the TaqMan MicroRNA Reverse Transcription Kit according to the manufacturer's protocol (Applied Biosystems, Foster City, CA, USA). Real-time PCR was performed with corresponding TaqMan primers and probes (Applied Biosystems, Foster City, CA, USA) in triplicate using the Opticon Real-time PCR system (Bio-Rad MJ Research, Waltham, MA, USA), according to the protocols provided by the manufacturers.

Western blot was performed as previously described [25] using an antibody against the human LIN28 protein (ab46020) (Abcam, Hong Kong). GAPDH protein was used as the loading control. The Western blot results were quantified with Quantity One (Bio-Rad Laboratories, Hercules, CA USA).

### Selection of SNPs in LIN28 for association studies

SNPs spanning a ∼21.5-kb region from 2 kb upstream to 0.5 kb downstream of the LIN28 gene were surveyed in the International HapMap Project database (HapMap Data Rel 27 Phase II+III, Feb 09, on NCBI B36 assembly). The International HapMap Project had genotyped a large number of SNPs in different populations and provided a set of tag SNPs (tSNPs) which could efficiently represent evolutionally linked genetic variants [28]. Using the Tagger Pairwise method with an *r^2^* cutoff of 0.8 and MAF cutoff of 0.05 in Han Chinese population, we selected five tSNPs (rs12122703, rs3811464, rs1247955, rs3811463, and rs6697410) which represent 68.8% (11/16) of the common SNPs in the LIN28 gene. The five excluded SNPs were located within the introns and they did not tag any other SNP. The selected tSNPs can tag all the SNPs located in the promoter and 3′ UTR of LIN28. Two SNPs (rs12122703 and rs3811464) were located in the 5′ flanking region (promoter) of the LIN28 gene, another two SNPs (rs3811463 and rs6697410) in the 3′ UTR, and the remaining SNP (rs11247955) was located in the intron (Figure S2A).

### Study populations and data collection for the case-control studies

The relationship between the common SNPs of LIN28 and the risk of breast cancer was investigated through two case-control studies in a stepwise manner. All the participants were genetically unrelated Han Chinese living in Shanghai City and its surrounding areas. The first hospital-based case-control study consisted of 1,004 patients with pathologically-confirmed primary breast cancer and who were consecutively recruited from the Department of Breast Surgery at Fudan University Shanghai Cancer Center (FDUSHCC) between January 2004 and June 2008. During the same period, 1,296 controls, matched to the cases by age and living regions, were enrolled from amongst women who had come to the Outpatient Department of FDUSHCC for breast cancer screening. In the validation study, we recruited an additional 511 patients through the same process between December 2008 and July 2009 and 645 control subjects who were frequency matched to the cases by age. These control subjects were from an on-going community-based breast cancer screening program that has been previously described [25]. Participants with a previous history of cancer (except breast cancer) and metastatic breast cancer were excluded from our study. All the controls were determined as cancer-free after comprehensive examinations.

Table S3 presents the detailed baseline characteristics of the subjects enrolled in this study. After finishing a written informed consent document, each participant was carefully interviewed to obtain epidemiological information. Each participant donated approximately 3–5 ml of peripheral venous blood, which was collected in tubes with either EDTA or heparin anticoagulant. This study was approved by the Ethics Committee of FDUSHCC. All clinical investigations have been conducted according to the principles expressed in the Declaration of Helsinki. The patients and controls in both the test set and the validation set were comparable in age (mean age was 48.2 years for the cases and 48.5 years for the controls in the first set; mean age was 48.6 years for the cases and 48.4 years for the controls in the validation set) and menopausal status (proportion of postmenopausal women was 37.1% for the cases and 24.7% for the controls in the test set; the proportion of postmenopausal women was 33.1% for the cases and 36.3% for the controls in the validation set). When compared with the controls, the patients in both sets were younger at menarche, and they had a higher body mass index (BMI) and fewer numbers of children (*P*<0.05). Furthermore, a significant number of these patients had a family history of first-degree relatives with breast cancer. These differences partially reflected some of the risk factors for breast cancer.

The clinicopathologic record of each patient was acquired via the Patient Database of FDUSHCC. The status of estrogen receptor (ER), progesterone receptor (PR), and human epidermal growth factor receptor 2 (HER2) was determined among patients with available data. The breast cancer IHC subtypes were defined as previously described [34], only our classification was based on the IHC results for the above mentioned three markers (ER, PR and HER2), and we merged luminal A with luminal B as the luminal subtype. Thus we defined breast cancer subtypes as: luminal (ER+ and/or PR+), basal-like (ER−, PR−, and HER2−), and HER2+/ER− subtype (HER2+, ER−, PR−).

### Genotyping

Genomic DNA was extracted from the blood leukocytes of the participants using Gentra's PureGene DNA Purification Kit (Gentra systems, Minneapolis, MN, USA). Genotyping was done mainly using the 12-plex SNPstream system (Beckman Coulter, Fullerton, CA, USA) at the Chinese National Human Genome Center at Shanghai. SNPs that could not be genotyped by this platform due to the inability to design appropriate primers or probes were genotyped by PCR and RFLP-based assays (Text S1, Figure S2B and Table S4). To ensure the reliability of the results, operators performing the genotyping assays were unaware of the disease status of each sample, and each batch of samples contained at least one positive control consisting of DNA samples with known genotype and two negative controls of pure water. Two researchers (K.D. Yu and A.X. Chen) independently examined the gel pictures of the RFLP assays. The assays were redone if there were inconsistencies regarding the results. Samples with disputable outcomes in the second test were directly sequenced. Ten percent of the samples were randomly selected for repeated RFLP assays, and the results were 100% concordant.

### Immunohistochemistry

The immunohistochemistry (IHC) assay was performed as previously described [35] using an antibody specific for LIN28 (ab46020) (Abcam, Hong Kong). The results were scored based on the percentage of tumor cells positively stained as follows: low expression required less than 15% of positive cells, moderate expression required 15–60% positive cells, and high expression required greater than 60% positive cells.

### Cell lines, LIN28 expression plasmid construct, and soft agar assay

The cell lines (MDA-MB-231, MCF7, T47D, SK-BR-3, BT-474, MCF 10A, and HEK 293T) were obtained from the American Type Culture Collection (ATCC) and maintained in complete growth medium as recommended by the distributor. Liquid nitrogen stocks were made upon receipt, and they were maintained until the start of each study. Morphology and doubling times were also regularly recorded to ensure maintenance of phenotypes. Cells were used for no more than 6 months after being thawed.

The CDS of human LIN28 was cloned into the pCDH-CMV-MCS-EF1-Puro expression vector (System Biosciences, Mountain View, CA, USA) and confirmed by direct sequencing (primers listed in Table S5). The empty or LIN28-CDS-fused pCDH vectors were then packaged into VSV-G-pseudotyped viral particles using the pPACKH1 Lentivector Packaging Kit (System Biosciences, Mountain View, CA, USA) according to the manufacturer's protocol. The unconcentrated viral supernatant was collected for infection.

MCF 10A cells stably transfected with LIN28 were acquired by infecting approximately 50,000 MCF 10A cells with 1 ml of unconcentrated viral supernatant, and they were selected in medium containing 3 ng/µl puromycin. Soft agar assays were performed as previously described [36]. Briefly, approximately 6,000 MCF 10A cells stably transfected with LIN28 or with empty vector were cultured in soft agar media (Promega, Madison, WI, USA) for three weeks before being stained with 0.05% crystal violet and photographed. The colony number was counted using Quantity One (Bio-Rad Laboratories, Hercules, CA USA).

### Power analysis

The program Quanto (http://hydra.usc.edu/gxe) was used to estimate the statistical power of our study. A dominant model was adopted and a range of allelic frequencies, odds ratios, prevalence of breast cancer observed in the studied population (25 in 100,000), and the sample size of 1,004 cases (accompanied by 1,296 controls) in the first case-control study were taken as the parameters. For alleles with minor allele frequencies of 15%–20%, our study has more than 95% power to detect alleles with ORs higher than 1.4 and more than 81% power to detect those with ORs of 1.3. The power drops for alleles with lower frequencies; for those with 10% frequency, the power to identify effects higher than 1.3 is 71%. When combining the two case-control studies together, we have more than 99% power to detect alleles with MAF higher than 15% and ORs of 1.4 and more than 93% power for alleles with ORs of 1.3 and MAF higher than 15%.

### Statistical analysis

HWE was tested by χ^2^ tests for each SNP locus. The associations between alleles and breast cancer risk were determined by Pearson's χ^2^ test, and the *P*-values were corrected using the 1,000-time permutation test [37] or Bonferroni correction. Logistic regression was used to analyze the associations between genotypes and breast cancer risk. The crude OR and OR adjusted for age, age at menarche, menopause status, number of birth, BMI and family history of breast cancer (in first- and second-degree relatives), along with 95% CI, were also determined. The distribution of rs3811463 genotypes in patients with different subtypes of breast cancer was analyzed by Pearson's χ^2^ test. Cochran-Mantel-Haenszel χ^2^ test was employed in association tests involving ordered variables. Student's t-test or Mann–Whitney U test was employed to compare continuous variables between two groups. One-way ANOVA or Kruskal–Wallis analysis was used to compare continuous variables among three or more groups. A *P*-value of no more than 0.05 was considered statistically significant. Statistical analysis was performed using Stata 10.0 (StataCorp, College Station, TX, USA) and SPSS Software version 12.0 (SPSS Inc., Chicago, IL, USA). The structure of the linkage disequilibrium (LD) block was constructed using Haploview 4.2 and showed in Figure S2C [37].

## Supporting Information

Figure S1 A, a schematic representation of the genomic locations of common SNPs and the target sites of microRNAs (miRNAs) in the 3′ UTR of the human LIN28 gene. According to the dbSNP database from National Center for Biotechnology Information (NCBI) and the International HapMap Project database, there are three SNPs with minor allele frequency (MAF) higher than 5% among Han Chinese in the 3′ UTR of the human LIN28 gene (rs4659441, rs3811463, and rs6697410). The SNPs and their locations, calculated from the start site of the 3′ UTR of LIN28, are presented above the horizontal axis. The target sites of miRNAs that might bind and regulate LIN28 according to TargetScanHuman (Release 5.1, http://www.targetscan.org/) are shown below the horizontal axis. The sequence, approximately 100-bp long and spanning from approximately positions +900–+1000 of the 3′ UTR of LIN28, as well as the locations of the target sites of *let-7* and rs3811463, are shown in detail above the horizontal axis. Among all of the predicted miRNA target sites, *let-7* is nearest to rs3811463. B, schematic representation of the luciferase reporter vectors. The control psiCHECK2 luciferase vector (top) and psiCHECK2 vectors contain a partial LIN28 3′ UTR sequence with either the T (middle) or C (bottom) allele at the rs3811463 locus. C, replication of the luciferase assay with increasing amounts of synthesized *let-7f*. T47D cells were transfected with increasing amounts of *let-7f* mimics, and the differential suppression of psiCHECK2:rs3811463T and psiCHECK2:rs3811463C was observed in a *let-7f* dose-dependent manner. D, real-time PCR evaluation of the levels of *let-7c* and *let-7d* in HEK-293T cells 48 hours after transfection with anti-*let-7c* and anti-*let-7d*. Both anti-*let-7c* and anti-*let-7d* could significantly suppress the level of the corresponding mature *let-7* family member.
(EPS)

Figure S2 A, candidate SNPs for genotyping. Five SNPs, spanning the ∼21.5-kb region from 2.0 kb upstream to 0.5 kb downstream of LIN28 gene, were selected for genotyping. Of those, two SNPs are located in the 5′ promoter region, and one is located in the introns, while the remaining two SNPs are located in the 3′ UTR of LIN28. B, genotyping of rs3811464 and rs3811463 by PCR- and RFLP-based assays. Above: Typical gel electrophoresis plots of the rs3811464G>A polymorphism. The gel electrophoresis was performed on 2.5% agarose gel at 120 V for 50 minutes. The main band from the rs3811464-GG genotype was approximately 170 bp in length, and the main band from the rs3811464-AA genotype was approximately 270 bp in length, while the rs3811464-GA genotype has both bands. Theoretically, there should be one more band of approximately 100 bp in length; however, this band was generally too weak to be detected. Below: Typical gel electrophoresis plots of the rs3811463 T>C polymorphism. The gel electrophoresis was performed on 2.5% agarose gel at 120 V for 40 minutes. The main band from the rs3811463-TT genotype was approximately 536 bp, and the main band from the rs3811463-CC genotype was approximately 379 bp. The heterozygote, rs3811463-TC, has both bands. C, pairwise linkage disequilibrium (LD) structure among selected SNPs in LIN28. Values within the boxes: pairwise LD values measured as D′ (×100). Red-to-white color gradient corresponds to higher to lower LD values; Solid red boxes are perfect correlations (D′ = 1). The direction of LIN28 transcription is from left to right. The graph was modified from the analysis results based on Haploview.
(EPS)

Table S1 Summary characteristics of the patients who donated normal breast tissue.
(DOC)

Table S2 Distribution of rs3811463 genotype in different subtypes of breast cancer.
(DOC)

Table S3 Summary characteristics of the participants.
(DOC)

Table S4 Primers and probes for SNP genotyping.
(DOC)

Table S5 List of primer sequences.
(DOC)

Text S1 RFLP assay.
(DOC)
