# Supplementary material for: Germline Genetic Variants Disturbing the Let-7/LIN28 Double-Negative Feedback Loop Alter Breast Cancer Susceptibility
Source: PLoS Genet. 2011 Sep 1;7(9):e1002259. doi: 10.1371/journal.pgen.1002259 (PMC3164678; doi:10.1371/journal.pgen.1002259)
Supplement: Table S1 — Summary characteristics of the patients who donated normal breast tissue. (DOC) [file pgen.1002259.s003.doc]

**Table S1. Summary characteristics of the patients who donated normal breast tissue**

| **Variables** | **rs3811463 genotype** | | ***P**** |
| --- | --- | --- | --- |
|  | **TT** | **TC** |  |
| **Age (year, mean ± SD)** | 49.2±11.4 | 50.6±9.3 | 0.62 |
| **Age at menarche (year, mean ± SD)** | 14.5±1.6 | 14.9±1.6 | 0.39 |
| **Menopause (*n*)** |  |  |  |
| No | 16 | 13 | 0.44 |
| Yes | 14 | 17 |  |
| **Family History (*n*)** ^§^ |  |  |  |
| Yes | 4 | 7 | 0.32 |
| No | 26 | 23 |  |
| **Estrogen receptor (ER) (*n*)** |  |  |  |
| Positive | 19 | 25 | 0.08 |
| Negative | 11 | 5 |  |
| **Progesterone receptor (PR) (*n*)** |  |  |  |
| Positive | 18 | 27 | 0.07 |
| Negative | 12 | 3 |  |
| **HER2 (*n*)** |  |  |  |
| Positive | 11 | 7 | 0.26 |
| Negative | 19 | 23 |  |
| **Histologic grade (*n*)** |  |  |  |
| G1 | 7 | 5 | 0.71 |
| G2 | 15 | 18 |  |
| G3 | 8 | 7 |  |
| **Tumor size (*n*)** ^‡^ |  |  |  |
| Tis | 5 | 2 | 0.69 |
| T1 | 8 | 9 |  |
| T2 | 15 | 17 |  |
| T3 | 2 | 2 |  |
| **Lymph node status (*n*)** ^‡^ |  |  |  |
| N0 | 21 | 15 | 0.26 |
| N1 | 3 | 8 |  |
| N2 | 4 | 6 |  |
| N3 | 2 | 1 |  |

* Two-sided χ^2^ test. *P* < 0.05 was considered statistically significant.

§ First- and second-degree relatives.

‡ According to American Joint Committee on Cancer (AJCC) TNM Staging System For Breast Cancer.

SD: standard deviation.
