# Supplementary material for: Germline Genetic Variants Disturbing the Let-7/LIN28 Double-Negative Feedback Loop Alter Breast Cancer Susceptibility
Source: PLoS Genet. 2011 Sep 1;7(9):e1002259. doi: 10.1371/journal.pgen.1002259 (PMC3164678; doi:10.1371/journal.pgen.1002259)
Supplement: Table S2 — Distribution of rs3811463 genotype in different subtypes of breast cancer. (DOC) [file pgen.1002259.s004.doc]

**Table S2. Distribution of rs3811463 genotype in different subtypes of breast cancer**

| Genotype | Subtypes of breast cancer (*n* = 918, %) ^†^ | | | Total | *P** |
| --- | --- | --- | --- | --- | --- |
|  | Luminal | HER2 | Basal-like |  |  |
| TT | 485 (75.1%) | 53 (8.2%) | 108(16.7%) | 646 (100%) | 0.26 |
| TG+GG | 201 (73.9%) | 16 (5.9%) | 55 (20.2%) | 272 (100%) |  |

* Two-sided χ^2^ test. P < 0.05 is considered statistically significant.

† Having missing data.
