# Supplementary material for: Germline Genetic Variants Disturbing the Let-7/LIN28 Double-Negative Feedback Loop Alter Breast Cancer Susceptibility
Source: PLoS Genet. 2011 Sep 1;7(9):e1002259. doi: 10.1371/journal.pgen.1002259 (PMC3164678; doi:10.1371/journal.pgen.1002259)
Supplement: Table S3 — Summary characteristics of the participants. (DOC) [file pgen.1002259.s005.doc]

**Table S3. Summary characteristics of the participants**

| **Variables** | **Test set, n (%)** | | **P^*^** | **Validation set, n (%)** | | **P^*^** |
| --- | --- | --- | --- | --- | --- | --- |
|  | **Patients (n = 1,004)** | **Controls (n = 1,296)** |  | **Patients (n = 511)** | **Controls (n = 645)** |  |
| Age (year) |  |  |  |  |  |  |
| ≤50 | 694 (69.12) | 853 (65.82) | 0.09 | 347 (67.91) | 412 (63.88) | 0.15 |
| >50 | 310 (30.88) | 443 (34.18) |  | 164 (32.09) | 233 (36.12) |  |
| Age at menarche (year) |  |  |  |  |  |  |
| ≤13 | 459 (45.72) | 414 (31.94) |  | 218 (42.66) | 226 (35.04) |  |
| >13 | 545 (54.28) | 882 (68.06) | <0.001 | 293 (57.34) | 419 (64.96) | 0.01 |
| BMI (kg/m^2^) |  |  |  |  |  |  |
| ≤23 | 456 (45.42) | 660 (50.93) |  | 227 (44.42) | 328 (50.85) |  |
| >23 | 548 (54.58) | 636 (49.07) | 0.01 | 284 (55.58) | 317 (49.15) | 0.03 |
| Menopause |  |  |  |  |  |  |
| No | 632 (62.95) | 846 (65.28) | 0.25 | 342 (66.93) | 411 (63.72) | 0.26 |
| Yes | 372 (37.05) | 450 (34.72) |  | 169 (33.07) | 234 (36.28) |  |
| Number of births^†^ |  |  |  |  |  |  |
| ≤2 | 917 (94.54) | 1,093 (91.77) | 0.01 | 464 (95.67) | 572 (92.71) | 0.04 |
| >2 | 53 (5.46) | 98 (8.23) |  | 21 (4.33) | 45 (7.29) |  |
| Family history ^§^ |  |  |  |  |  |  |
| Yes | 146 (14.54) | 106 (8.18) | <0.001 | 64 (12.52) | 55 (8.53) | 0.03 |
| No | 858 (85.46) | 1,190 (91.82) |  | 447 (87.48) | 590 (91.47) |  |

* Two-sided χ^2^ test. P < 0.05 is considered statistically significant.

† Having missing data.

§ First- and second-degree relatives.
