# Supplementary material for: Germline Genetic Variants Disturbing the Let-7/LIN28 Double-Negative Feedback Loop Alter Breast Cancer Susceptibility
Source: PLoS Genet. 2011 Sep 1;7(9):e1002259. doi: 10.1371/journal.pgen.1002259 (PMC3164678; doi:10.1371/journal.pgen.1002259)
Supplement: Table S4 — Primers and probes for SNP genotyping. (DOC) [file pgen.1002259.s006.doc]

**Table S4**. Primers and probes for SNP genotyping

| **SNP** | **Primer/Probe** | **Sequence** |
| --- | --- | --- |
| **SNPstrem** |  |  |
| rs12122703 | Sense | CCCACTCATCCGGTCTCT |
|  | Antisense | AATTCTCACGCTGTGGCTC |
|  | Probe | GGCTATGATTCGCAATGCTTGAAATACACATGTGCCCACATCCGC |
| rs11247955 | Sense | CAATGGATTATTAATTTTTATAGCAGC |
|  | Antisense | CAAGCAGATGGATCAGAGGA |
|  | Probe | GCGGTAGGTTCCCGACATATCTATTGATTTCTCCATATATTCTCA |
| rs6697410 | Sense | TTCCCTTCTCCTTTCCCTG |
|  | Antisense | AGTTATCGGAAGGGCAAAAA |
|  | Probe | ACGCACGTCCACGGTGATTTTGAATAAATAAAGACTTATTGGTAC |
| **RFLP** |  |  |
| rs3811464 | Sense | AGGCAAAGGGTTGGTTCGG |
|  | Antisense | CACCTGTATCTGCTTTGGGGAC |
| rs3811463 | Sense | TTTATTTGCTCCCTTGGATACTGC |
|  | Antisense | AACCCTCACCTAATCTTGGCTTG |
