# Supplementary material for: Germline Genetic Variants Disturbing the Let-7/LIN28 Double-Negative Feedback Loop Alter Breast Cancer Susceptibility
Source: PLoS Genet. 2011 Sep 1;7(9):e1002259. doi: 10.1371/journal.pgen.1002259 (PMC3164678; doi:10.1371/journal.pgen.1002259)
Supplement: Table S5 — List of primer sequences. (DOC) [file pgen.1002259.s007.doc]

**Table S5. List of primer sequences**

| **Purposes** | **Primer type** | **Sequence (restriction endonuclease)** | **Length (bp)** | **Tm (℃)** |
| --- | --- | --- | --- | --- |
| psiCHECK2-LIN28 | Sense | CCGCTCGAGCGGTATTTATTTGCTCCCTTGGATACTGC (Xhol) | 885 | 55 |
|  | Antisense | ATAAGAATGCGGCCGCTAAACTATTGATGTCACAGCGTGTAAACGAAAC (NotI) |  |  |
| pCDH-LIN28 | Sense | GGAATTCCGCTTCTTCTCCGAACCAA (EcoRI) | 788 | 57 |
|  | Antisense | CGGGATCCCGTCTCCACTCTGCCTGCTC (BamHI) |  |  |
| LIN28 real-time PCR | Sense | CAGGTGCTAGAAACTTTATGTCA | 139 | 52 |
|  | Antisense | AGGCTTTCCTACCCTCCC |  |  |
| GAPDH real-time PCR | Sense | GGGAGCCAAAAGGGTCATCATCTC | 353 | 60 |
|  | Antisense | CCATGCCAGTGAGCTTCCCGT |  |  |
