# Supplementary material for: Germline Genetic Variants Disturbing the Let-7/LIN28 Double-Negative Feedback Loop Alter Breast Cancer Susceptibility
Source: PLoS Genet. 2011 Sep 1;7(9):e1002259. doi: 10.1371/journal.pgen.1002259 (PMC3164678; doi:10.1371/journal.pgen.1002259)
Supplement: Text S1 — RFLP assay. (DOC) [file pgen.1002259.s008.doc]

**Text S1**

**RFLP assay**

Two single nucleotide polymorphisms (SNPs) (rs3811463 and rs3811464) could not be genotyped using the 12-plex SNPstream system (Beckman Coulter) due to our inability to design specific and perfect probes or primers, and were genotyped by PCR- and RFLP-based assays. For the detection of rs3811464, the PCR products (270 bp) were digested with adequate NlaIV (New England BioLabs) at 37℃ for 6 hours. The products containing the rs3811464A allele could not be cleaved, while the rs3811463-G allele could be digested into 170- and 100-bp fragments (**Figure S2B**). Similarly, the PCR products (536 bp) containing rs3811463 were digested with XcmI (New England BioLabs) at 37℃ for 5 hours, and the rs3811463T allele could not be digested, while the rs3811463C allele could be cleaved into two bands of 379 and 157 bp (**Figure S2B**). The primers are available in **Tables S4 and S5**.
